# The link between exposure to violence and psychological distress among middle-aged Muslims in Israel: the role of gender

**DOI:** 10.3389/fpubh.2024.1382053

**Published:** 2024-06-05

**Authors:** Khalil Iktilat, Michal Isacson, Roy Tzemah-Shahar, Maayan Agmon

**Affiliations:** ^1^Department of Gerontology, Faculty of Health and Social Welfare, University of Haifa, Haifa, Israel; ^2^Ramat Gan Academic College, Ramat Gan, Israel; ^3^The Cheryl Spencer Institute for Nursing Research, Faculty of Health and Social Welfare, University of Haifa, Haifa, Israel

**Keywords:** exposure to violence, psychological distress, middle-aged Muslims, mental health, healthy aging

## Abstract

**Introduction:**

To date, it is still unclear if exposure to violence affects psychological distress in middle-aged adults and if the effects are gender specific. This age group is of special interest as it is at the onset of the aging process and is often overlooked or understudied in scholarly research. Specifically, targeted research on middle-aged Muslims living in Israel, a unique population exposed to increasing violence, is lacking.

**Methods:**

We examined the relationship between exposure to violence and psychological distress in a cohort of 363 middle-aged adults (223 women) from three Muslim villages in northern Israel, collecting data on violence exposure (Screen for Adolescent Violence Exposure (SAVE) questionnaire), psychological distress (Kessler 6 Psychological Distress questionnaire), and other demographic characteristics including education level and socioeconomic status. We used this data to answer two questions: (1) is exposure to violence a predictor of psychological distress in middle-aged Muslims, and (2) does the relationship between exposure and distress differ between men and women?

**Results:**

We revealed a positive link between exposure to violence and psychological distress (*β* = 0.145, *p* = 0.017) when controlling for gender, age, education level, and socioeconomic level.

**Discussion:**

Despite previous evidence of gender-based differences in this interplay in younger cohorts, we did not find a significant interaction between gender and the violence exposure-psychological distress interplay. Our findings are some of the first to focus on middle-aged individuals and show that both men and women exhibit connections between exposure to violence and psychological distress when considering covariates. This research provides insights that can be used when planning community-wide interventions and treatment schemes to support healthy aging.

## 1 Introduction

Psychological distress is an essential component of mental health that significantly impacts quality of life (1). It is defined as persistent experiences of emotional anguish, discomfort, or disquiet (2) and is usually quantified by one or more validated, subjective questionnaires. Psychological distress affects 5–27% of the total population (2–7), and depression, anxiety, and post-traumatic stress disorder (PTSD) have all been reportedly associated with such distress (8). When untreated, psychological distress has also been closely linked to physiological disorders (9) such as immune system dysfunction and chronic diseases, such as cardiovascular illnesses, diabetes, hypertension, and some cancers (10–13), with a mortality rate 2–3 times greater in psychologically distressed individuals than the general population (12). Accordingly, when untreated, psychological distress can reduce lifespan by 10–20 years (12), emphasizing the importance of considering psychological distress when planning for healthy aging.

Several risk factors for psychological distress were described and can be largely divided into individual and community variables, though there can be some overlap between the two. This division is often useful when considering the types of interventions suited for a specific individual or community. On the individual level, factors such as a history of trauma (including direct violence), childhood adversity, low education, genetic predispositions, preexisting mental health conditions, and even life stage/age can increase susceptibility to psychological distress (14). Adolescents (7, 14–19) are particularly susceptible to psychological distress (15–18), but as individuals age, the links between individual risk factors and psychological distress are more poorly understood with some studies presenting conflicting results. For example, several studies found that psychological distress is associated with lower levels of education in adults [e.g., (7, 19)] but there is also evidence that intermediate schooling can increase distress in adults (20). Additionally, community-wide factors, including socioeconomic disparities, limited access to healthcare, and exposure to violence, have also been implicated in psychological distress (21). Indeed, people who are frequently exposed to violence typically exhibit a range of emotional and cognitive responses, including hypervigilance, intrusive thoughts, and emotional numbing (22, 23), all of which are associated with psychological distress (24). Furthermore, research has illuminated the relationships not only between experiencing violence but also witnessing it, and the subsequent development of psychological distress (25, 26). The research body regarding effects of community violence is also growing (27–30). One such example is a study of the effects of crime-related traumatic events on psychiatric disorders among over 5,000 individuals aged 18+, which found that as exposure to urban violence increased, severity of psychiatric disorders also increased. Over 40% of respondents exposed to 3 or more traumatic events reported moderate to severe psychiatric disorders and for those exposed to 1–2 traumatic events, this percentage was around 20% (30). Studies in middle-aged adults are still lacking, though.

Understanding the relationship between exposure to violence and psychological distress is crucial for prioritizing health and overall quality of life as well-planned interventions might need to be adjusted to fit specific community needs (31), particularly when combatting violence locally is challenging. An example of a high-violence environment is that occurring in many Muslim villages in Israel. While this sub-population is relatively modern and egalitarian, over the past decade, members of Muslim communities have witnessed a troubling increase in violent incidents; in 2018 alone, there were 71 homicides reported within this sub-population and in 2022, the number jumped to 121. One year later, in 2023, at the time of this writing, the number of homicides reported in the Arab community more than doubled from the previous year, to 244 (32). This increase in violence is at the forefront of governmental discussions, yet national efforts to reduce it have had minimal effects. Importantly, when examining violence throughout Israel (2021), over 40% of incidents were in non-Jewish communities (involving non-Jewish individuals), but the proportion of the non-Jewish population in Israel is only 0.20, especially in the northern part of the country, the place where the research was carried out and where 58% of the Muslim population lives (33, 34). This highlights the disproportionate exposure to violence among Israeli Muslims. This surge in violence has had a profound impact on their daily lives, making residents apprehensive about moving around in their local communities and neighborhoods. There has also been an increase in mental illness referrals to social workers, an increase that highlights the growing need for targeted interventions in this society (29).

Although the research body on the connection between violence and psychological distress is growing, middle-aged populations are largely understudied. Currently most research focuses on this relationship in youth (35–38) and study of this unique population, at the onset of aging, could reveal nuances in the violence-distress dynamic. More specifically, there is currently limited research on the link between violence exposure and psychological distress among Israeli Muslims, despite a surge in their exposure to violence. Middle-aged individuals represent a large proportion of the Muslims living in Israel, and they are an important target when considering healthy aging. One study (women >15 years, median age 22.6) found that violence exposure increases the risk of PTSD and anxiety, especially in areas with high violence levels [assessed by national health surveys, (39)]. The researchers also found that men, but not women, exposed to violence were more likely to experience anger management problems and aggressive behavior (39) highlighting differences in outcomes between the two genders following exposures to violence in a younger adult cohort. This difference in distress response to violent environments is interesting because research in youth [mean 14.15 years (time 1), 16.7 years (time 2)] supports heightened responses in females rather than males, possibly as a result of behavioral plasticity at this age or societal norms and the type of violence experienced (40). On the whole, these contradictory findings suggests that there may be a complex relationship between violence exposure, age, and gender on development of psychological distress (41) particularly as individuals age, and more research is needed across the lifespan and throughout the aging process.

The main objective of this study was to investigate the association between exposure to violence and psychological distress and identify gender-specific patterns in middle-aged Muslims in northern Israel, while controlling for differences in education, age, and socioeconomic status (42), factors that were previously linked to psychological stress. We hypothesized that (1) exposure to violence is positively associated with psychological distress among middle-aged Muslims; and (2) the connection between exposure to violence and distress will be different in men and women.

## 2 Materials and methods

### 2.1 Study design, cohort description, and study parameters

**Figure 1:**
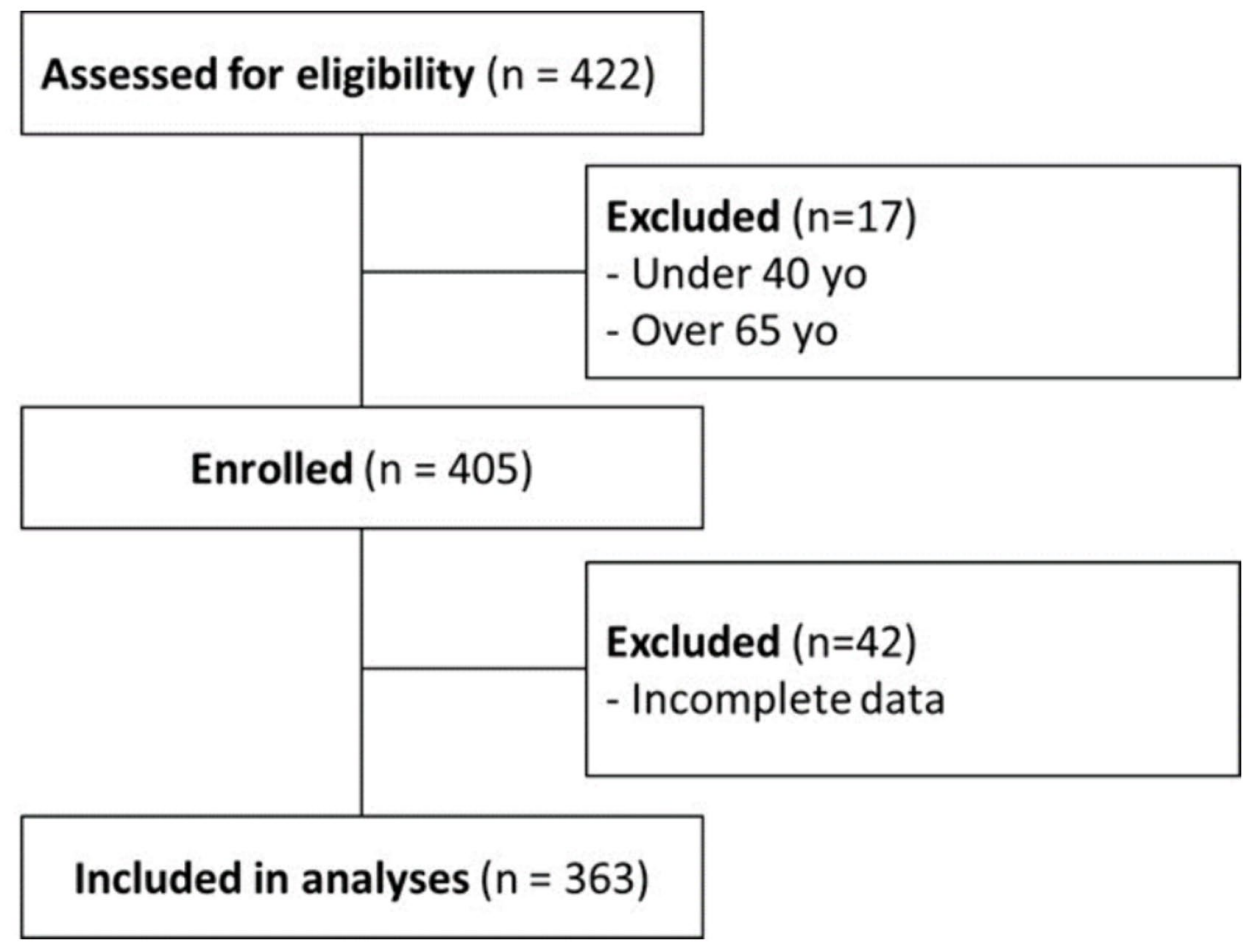
Flow chart of participant recruitment and enrollment.

This study is a part of larger cross-sectional study that explores the relationships between the environment and biological aging among middle-aged Muslims in Israel. Participants were recruited in three Muslim villages in northern Israel between March and September 2021 through a website/mobile app, posters in public buildings, and by word of mouth. The inclusion criterion was age-based: 40 to 65 years old; exclusion criteria were (1) history of neurological disorders (e.g., stroke or other neurological disorders) or major visual or hearing deterioration and (2) acute medical complaints (e.g., back pain or other acute illness; Figure 1). The study was approved by the Ethics Committee of the Faculty of Social Welfare and Health Science at the University of Haifa, Israel under approval number: 237/21. Data collection was performed in an hour-long session in which participants received an explanation about the study, signed informed consent, and provided the below-described measures.

#### 2.1.1 Psychological distress

Psychological distress was assessed using the Kessler 6 Psychological Distress Scale questionnaire containing six items focusing on psychological distress and emotional state (43). Each question was rated using a five-point Likert scale ranging from 1 (“never”) to 5 (“always”), and then distress was characterized as the mean number of points across all questions. We chose this questionnaire because it is a broadly used, validated method [e.g., (44, 45)], and it also shows high repeatability in diverse cohorts, including in a study in an Arab population similar to ours (Cronbach’s α = 0.81) (46).

#### 2.1.2 Exposure to violence

The Screen for Adolescent Violence Exposure (SAVE) questionnaire was used to quantify exposure to violence (47). It consists of 30 items describing different violent situations, such as “I saw someone carrying a gun,” or “I heard someone being beaten,” divided into sub-sections, with each presenting a different type of exposure, such as exposure to traumatic violence or verbal or physical violence. For this study, the original questionnaire was translated into Hebrew; cross-translation was performed by the research team, following the recommended guidelines (48), and respondents were asked to rate each situation on a five-point Likert scale ranging from 1 (“never”) to 5 (“always”), representing the degree of exposure. Each participant’s exposure to violence was obtained by calculating the mean of the number of points per question. The original SAVE questionnaire was previously validated both objectively with local crime data and theoretically, and was found to be highly reliable; in the previous validation the SAVE results were significantly correlated with high and low violence based on local data, and the mean Cronbach’s α for internal consistency was 0.81 across all sub-fields examined (47).

#### 2.1.3 Demographic data

Demographic data (e.g., age, education level, socioeconomic status) and health behaviors (e.g., smoking status) were collected using self-administered questionnaires. Socioeconomic status was rated using a single question focusing on subjective income level in comparison to the national average, using a five-point Likert scale ranging from 1 (“significantly lower than the average”) to 5 (“significantly higher than the average”). Education level was measured as having completed at least an associate’s degree (yes/no) and also as years of schooling. These variables were considered because they have previously been found to have associations with the development of psychological distress. Further, we examined their correlations with psychological distress and with exposure to violence and included significant factors in our models.

### 2.2 Statistical analysis

Formal analysis of results was conducted using IBM SPSS v.27.0.0.0; the level of significance was set at *p* < 0.05. Variables are presented as mean ± SD (standard deviation) and outliers (beyond two standard deviations of the mean) were excluded. Comparisons between men and women were made using t-tests or Mann–Whitney U-tests, as appropriate, for continuous data, and Fisher exact tests for categorical data. Bivariate correlations were examined to determine which demographic factors were correlated with psychological distress and violence exposure (Pearson correlation coefficient) and then controlled for in linear regression models.

To address our first hypothesis, that exposure to violence is positively associated with psychological distress among middle-aged Muslims, a linear regression model was used to assess the association between psychological distress (response) and exposure to violence (independent variable) with models adjusted for age, gender, education level, and socioeconomic status (based on findings from bivariate correlation analyses). To address our second hypothesis, that the connection between exposure to violence and psychological distress differs between men and women, we used a linear regression, like above, but with an additional interaction term between gender and violence exposure.

## 3 Results

### 3.1 Cohort characterization

**Figure 2:**
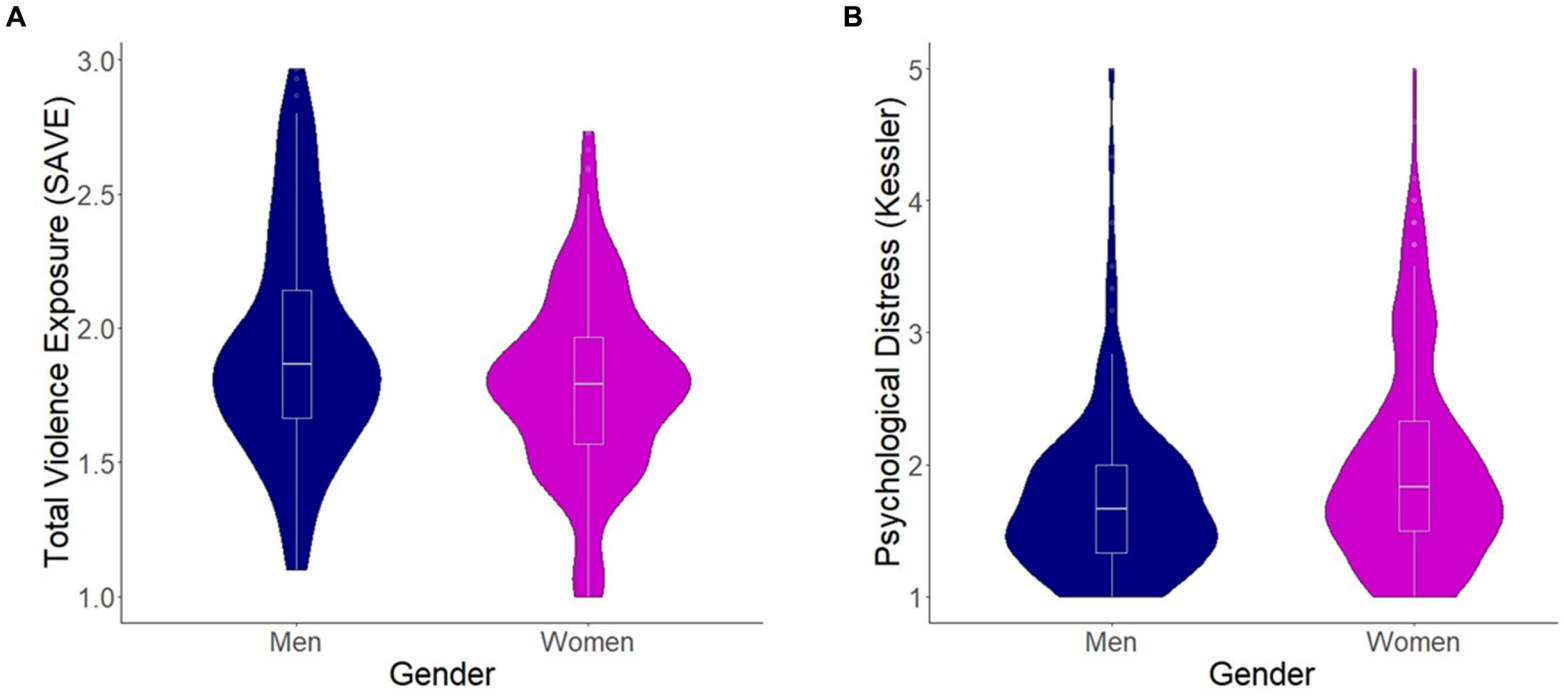
Comparison of violence exposures and psychological distress levels among men and women. **(A)** The average sub-category scores of the Screen for Adolescent Violence Exposure (SAVE) questionnaire, compared between men and women using a *t*-test (*p* = 0.01). **(B)** The average sub-category scores of the Kessler-6 (psychological distress) questionnaire, compared between men and women using a *t*-test (*p* = 0.003). Violin plots represent distributions of the data and box plots represent the median, 1st, and 3rd quartiles of the data. Blue, men, pink, women.

A total of 363 participants (223 women, 140 men), aged 50.26 ± 6.29 (40–65 years) were included in the final analysis. Demographics and distributions of the main variables are presented in Table 1. While overall exposure to violence was high [98.6% of participants reported at least some exposure (mean SAVE score greater than 1, indicating at least some violence exposure); Figure 2A], men reported significantly more violence exposure than women (*p* = 0.01); women reported significantly more psychological distress (*p* = 0.003; Figure 2B).

**Table 1:** Descriptive statistics of the middle-aged Muslim cohort.

	Total cohort	Men	Women	*p* value
Sample size (%)	363	140 (38.6%)	223 (61.4%)	–
Age	50.26 ± 6.29 (40–65)	51.62 ± 6.68 (40–65)	49.41 ± 5.89 (40–64)	0.001^1^
Income^2^	2.57 ± 1.1 (1–5)	3.12 ± 1.04 (1–5)	2.19 ± 0.97 (1–4)	<0.001^3^
SAVE^2^	1.84 ± 0.37 (1–2.97)	1.92 ± 0.41 (1.1–2.97)	1.78 ± 0.34 (1–2.73)	0.01^1^
Kessler total score^2^	1.90 ± 0.73 (1–5)	1.76 ± 0.64 (1–5)	1.98 ± 0.78 (1–5)	0.003^1^
Education (category)	(*N* = 320)	(*N* = 124)	(*N* = 196)	0.006^4^
Academic	46.3%	55%	40.8%	
Non-academic	41.9%	33.6%	47.1%	
Education (years)	14.93 ± 4.95	15.01 ± 4.17	14.88 ± 5.07	0.853
BMI	30.02 ± 4.83 (19.54–56.51)	29.99 ± 4.04 (21.50–43.12)	30.04 ± 5.28 (19.54–30.04)	0.01^1^
Active smokers	21.7%	42.9%	8.5%	<0.001^4^

Characteristics of the cohorts were compared between men and women with relevant statistical tests. Data are presented as mean ± SD (range), SAVE: violence exposure metric, Kessler: psychological distress measure. ^1^*T*-test; ^2^Likert scale: 1–5, 5 highest; ^3^Mann–Whitney U Test; ^4^Fisher exact test.

### 3.2 Bivariate correlates of psychological distress

**Figure 3:**
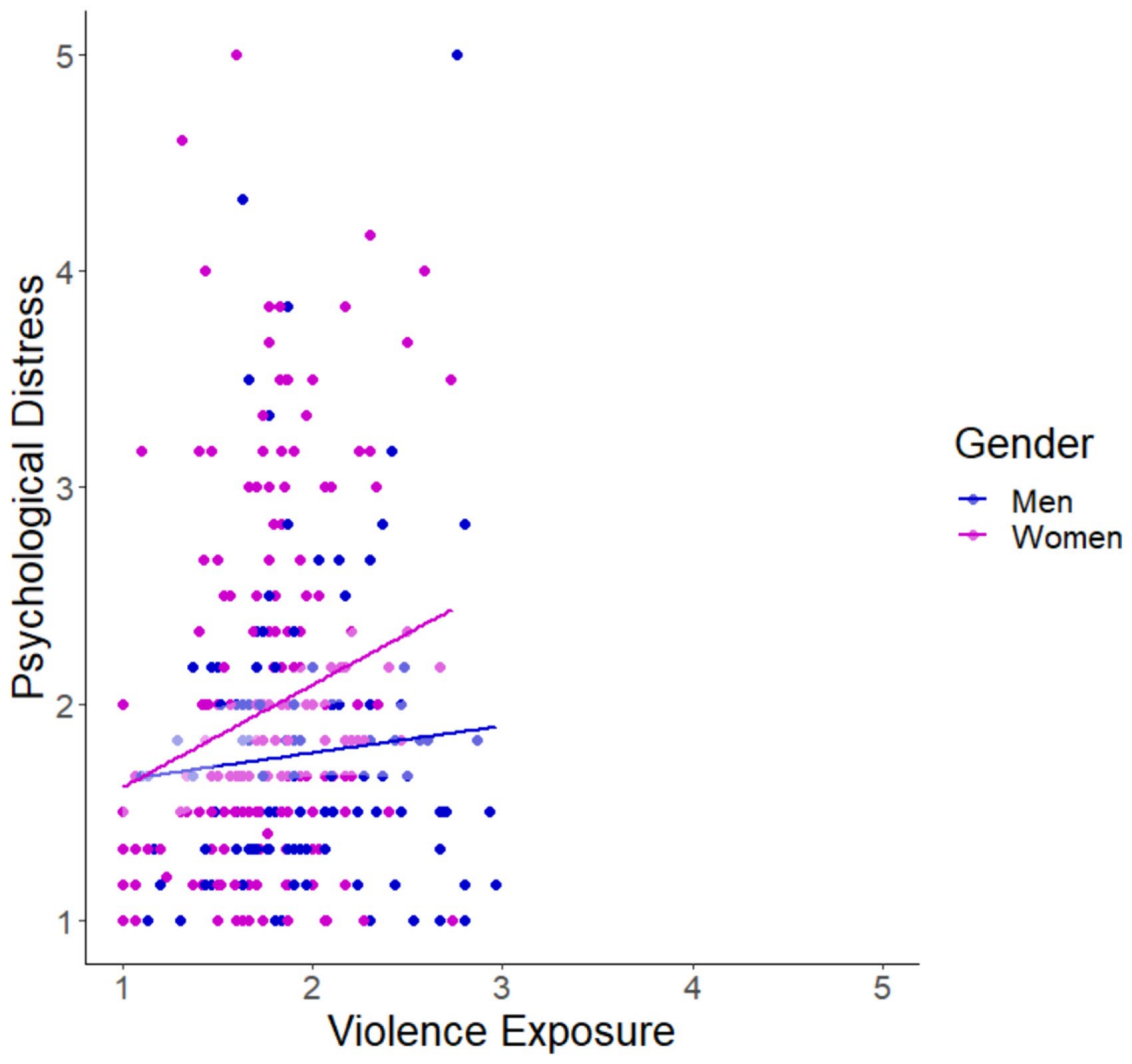
Gender-specific relationship between violence exposures and psychological distress levels in a middle-aged Muslim cohort. Violence exposure (1–5: Screen for Adolescent Violence Exposure (SAVE) questionnaire) and psychological distress levels (1–5: Kessler-6) for men (blue; *r* = 0.080, *p* = 0.358) and women (pink; *r* = 0.208, *p* = 0.002) with linear trendlines. A regression model (Table 3) revealed that there was a significant association between violence exposure and psychological distress for the total cohort when controlling for covariates, but no between-sex differences were observed.

Psychological distress was significantly and positively correlated with exposure to violence (Table 2; Figure 3; Pearson correlation: *r* = 0.124, *p* = 0.05). Age (*r* = −0.151, *p* = 0.01) and socioeconomic status (*r* = −0.128, *p* = 0.05) were also significantly correlated with psychological distress, though for both variables, the correlation was negative. There was also a significant relationship with gender (Table 1, *t*-test: *p* = 0.003) and with completion of an academic degree (Table 2, *t*-test: *p* = 0.046) on psychological distress. These four variables (age, socioeconomic status, gender, and education) were also significantly associated with levels of exposure to violence (Tables 1, 2). Therefore, they were included as covariates in our linear models (below).

**Table 2:** Bivariate correlations of study parameters with psychological distress.

	Pearson’s *r*	*p* value
**Psychological distress (Kessler 6)**		
SAVE	0.124	0.020
Age	−0.151	0.004
Socioeconomic status	−0.128	0.020
Education*	2.004	0.046
**Violence exposure (SAVE)**		
Kesser-6	0.124	0.020
Age	−0.115	0.015
Socioeconomic status	0.179	0.001
Education*	−3.138	0.002

Kessler 6: measure of psychological distress. SAVE: measure of violence exposure, *n* = 363. The relationships between education (binary variable: advanced degree – yes/no) and distress and violence exposure, respectively, were examined with a *t*-test and *t*-statistic is provided instead of Pearson’s *r*.

Following bivariate correlation analysis, we aimed to answer our first research question. A linear regression model to better understand the link between violence exposure (SAVE score) and psychological distress (Kessler score) was built controlling for education level, socioeconomic status, and age (Table 3). Exposure to violence was found to be significantly and positively correlated with psychological distress (*β* = 0.145, *p* = 0.017), as was gender (*β* = 0.132, *p* = 0.045).

**Table 3:** Regression model for whole cohort.

	Independent variables	Standardized coefficients beta	*p* value
**Dependent variable**			
Psychological distress	1. Gender	0.132	**0.045** ^**1**^
	2. Education	−0.121	0.063
	3. Socioeconomic status	−0.25	0.729
	4. Age	−0.111	0.063
	5. Total SAVE score	0.145	**0.017** ^**1**^

Model adjusted for gender, education, socioeconomic status, age, total SAVE score. *N* = 363. The R^2^ of the model was 0.067.

1 Significant findings are in bold.

Toward answering our second research question, we ran a linear regression model, like above, but with an interaction term between gender and violence exposure. This interaction was not significant (*β* = 0.073, *p* = 0.360), suggesting that the positive relationship between violence exposure and psychological distress that we observed in the general population is in fact representative of both men and women.

## 4 Discussion

In this study, we examined the relationships between violence exposure and psychological distress in middle-aged Muslims living in Israel who have been exposed to increasing violence over the past decade. Both men and women in our study population were exposed to violence with over 98.6% of participants reporting at least some exposure. Although men reported increased exposure compared to women, the latter had increased self-reported psychological distress (Table 1). We found a positive correlation between exposure to violence and psychological distress (Tables 2, 3) but did not find an interaction between gender, exposure to violence, and psychological distress (interaction effect) when considering covariates in our full linear model.

Our study emphasizes the substantial link that exposure to violence may have on a person’s mental health and suggests that the link is not gender-based in middle aged adults. When considering only bivariate analyses (Figure 3), we found that women but not men had significantly correlated levels of violence exposure and psychological distress, but in full models including relevant covariates (described in the Methods), this relationship was not maintained. Our findings are in line with a previous study examining the complex dynamics pertaining to the gender-specific impacts of environmental exposures, including political violence, on psychological distress which found that women were more impacted than men [assessed by the 17-item PTSD Symptom Scale Interview format (PSS-I)], but that inclusion of a covariate in the model, namely subjective health, completely mediated the effect of gender on psychological distress (49). Several studies show different psychological responses to violence among males and females, but they are in the context of adolescents [assessed by: Trauma Symptom Checklist for Children (TSCC) or Anxiety Module of the Diagnostic Interview Schedule for Children, Fourth Edition (DISC-IV), (40, 50, 51)]. One such study found that adolescent girls exposed to potentially traumatic events (including violent events) may be more vulnerable than boys to experiencing certain trauma-related symptoms, particularly dissociation. These findings suggest that gender specific pathways to trauma-related psychopathology might be limited to this sensitive period in life. One implicated pathway in the adolescent pattern of gender-based distress differences is that of the hypothalamic–pituitary–adrenal (HPA) axis which, when dysregulated in boys leads to hyperarousal (fight or flight), but in girls can lead to decreased responsiveness (dissociation) (40).

Similarly, neurobiological changes underlying gendered violence-distress patterns have been preliminarily studied in developing children and school-aged youth [assessed by thematic content analysis of individual interviews or modified World Health Organization’s Health Behavior in School-aged Children Survey—HBSC, (52, 53)]. According to Peverill et al. (54), children who have experienced violence exhibit altered amygdala connectivity, which is linked to heightened responses to fear stimuli and decreased emotional regulation. This same study found alterations in stress hormones in connection to both violence exposure and psychological distress [assessed by Diagnostic Interview Schedule for Children, Version IV, (54)]. Alterations in cortisol levels have also been associated with elevated risks of anxiety, depression, and PTSD [e.g., (55)]. The combination of the absence of relevant neurobiological findings regarding violence exposure and psychological distress in adults in the body of literature and our findings that there are no gender-based differences in the relation between violence exposure and psychological distress among a cohort of highly exposed middle-aged individuals could suggest that as individuals age, gendered differences in the violence-distress interplay decrease. It is important to note, however, that the length of violence exposure was not examined here, and persistent vs. acute exposures could be associated with different, even gendered, patterns of psychological distress.

The impact of exposure to violence on men’s psychological state has been the subject of a number of studies. While some found no relationship between the two [assessed by GHQ-12 or national health interview survey of wellbeing, (56, 57)], others showed violence to be a significant predictor of psychological distress [assessed by International Childhood Disorders-8, Strengths or Difficulties Questionnaire (SDQ) and PTSD Symptoms Scale (PTSDSS), (58–60)]. Our bivariate findings are in line with the former category, though when considering covariates, the relationship between violence and distress becomes significant. A recent study of over 6,000 18-34-year-olds in Sweden found that while men’s exposure to violence was two times as high as women’s, the odds ratio (OR) of poor mental health was 2.66 in women and 1.12 in men, and this was maintained even after including covariates (56). Another study of 18-24-year-olds from Denmark found that while men were more likely to be exposed to violence than women (OR = 3.2), only exposed women were more likely to report anxiety and depression compared to their unexposed counterparts; no such relationship was found between exposed and unexposed men. In this study, a full model including covariates was not presented (57). A third study, from Israeli, based on data from 2007 of nearly 1,200 Palestinian adults with a mean age of 35.03 ± 12.67 found gender, namely being a woman, to be significantly and positively associated with psychological distress levels as well (49). Importantly, these three studies focused on younger cohorts than our study population (mean age: 50.26 ± 6.29). Further exploration into age-associated, gendered effects on violence exposure and psychological distress is needed as men may not respond to questions candidly in certain age classes because they do not want to seem weak or because they are taught from a young age that being tough and using violence is part of being a man. Another possibility is that men might not feel as powerless as women when it comes to violence. These could also be interrelated, and we should explore their psychological impacts further.

One of Arab Israelis’ biggest public health issues is psychological distress, with a previous study citing 35% of the population reporting symptoms of distress [assessed by GHQ-12, (61)], which is further supported in our research. While reducing violence exposure is a primary goal, early interventions and targeted treatment for distress (53, 62) will also help reduce the burden of violence-related distress, ultimately fostering a safer and healthier community (63). Accordingly, the stigma surrounding seeking support services should also be addressed. Likewise, in Israel, as in many other regions, there is also a growing need to educate individuals about the potential harm of repeated exposure to violent incidents in the news and on social media toward reducing vicarious trauma (64). By promoting media literacy and responsible media consumption (validated sources, limited duration), individuals can gain the tools to critically assess and interpret the information they encounter, thus reducing the psychological burden (53). This comprehensive approach of early intervention coupled with behavioral changes can contribute to creating a more informed and resilient society in the face of escalating violence (65).

## 5 Limitations and future directions

While research on the connection between violence exposure and psychological distress has advanced significantly, in large part, from the work presented here, many questions remain, particularly surrounding the middle-aged population. For instance, our research did not examine the bio-physiological processes by which exposure to violence results in psychological distress (66). Additionally, interactions with other elements—like employment, culture, religion, and ethnicity—should also be considered when studying psychological distress in middle-aged populations. Lastly, the number of men and women included in the study was not equal, and they were not exposed to the same levels of violence, based on self-reporting, which could complicate understanding of the genders’ violence-distress interplay. Future insights on this topic can be expanded using longitudinal studies and more complex statistical models, toward gaining a comprehensive understanding of the factors influencing and mediating psychological distress in middle-aged individuals. Further study of this relationship will allow for the creation of more customized and effective interventions (67).

## 6 Conclusion

In conclusion, our research on middle-aged Muslims living in Israel revealed a significant positive relationship between exposure to violence and psychological distress among females and males when considering covariates. This contrasts with the gendered relationship observed among younger cohorts. Further uncovering age-related mediators of gendered violence-distress interplay can assist in building targeted interventions to prevent violence-associated psychological distress across the lifespan, with a focus on improving the aging process in light of the Muslim community’s increasing violence exposures.

## Data availability statement

The raw data supporting the conclusions of this article will be made available by the authors, without undue reservation.

## Ethics statement

The study was approved by the Ethics Committee of the Faculty of Social Welfare and Health Science at the University of Haifa, Israel under approval number: 237/21. Participants provided written informed consent to participate in this study.

## Author contributions

KI: Writing – original draft, Visualization, Project administration, Methodology, Investigation, Formal analysis, Data curation, Conceptualization. MI: Writing – review & editing, Methodology, Conceptualization. RT-S: Writing – review & editing, Visualization, Formal analysis, Data curation. MA: Writing – review & editing, Visualization, Supervision, Project administration, Methodology, Conceptualization.
